# In Vitro Anticancer and Radiosensitizing Activities of Phlorethols from the Brown Alga *Costaria costata*

**DOI:** 10.3390/molecules25143208

**Published:** 2020-07-14

**Authors:** Olesya S. Malyarenko, Tatiana I. Imbs, Svetlana P. Ermakova

**Affiliations:** G.B. Elyakov Pacific Institute of Bioorganic Chemistry, Far-Eastern Branch of the Russian Academy of Sciences, 159, 100-let Vladivostoku Prospect, 690022 Vladivostok, Russia; tatyanaimbs@mail.ru (T.I.I.); ermakova@piboc.dvo.ru (S.P.E.)

**Keywords:** phlorotannins, phlorethols, anticancer activity, colorectal cancer, radiosensitizer, radiotherapy

## Abstract

The anticancer and radiosensitizing effects of high-molecular-weight phlorethols **CcPh** (Mw = 2520 Da) isolated from the brown algae of *Costaria costata* on human colorectal carcinoma HCT 116 and HT-29 cells were investigated. Phlorethols **CcPh** possessed cytotoxic activity against HT-29 (IC_50_ = 92 μg/mL) and HCT 116 (IC_50_ = 94 μg/mL) cells. **CcPh** at non-toxic concentrations inhibited the colony formation in colon cancer cells and significantly enhanced their sensitivity to low non-toxic X-ray irradiation. The combinatory effect of radiation and **CcPh** was synergistic (Combination index < 0.7). Algal phlorethols might be prospective candidates as radiosensitizers to improve the scheme of radiotherapy.

## 1. Introduction

Radiation therapy is traditionally considered one of the most effective methods for influencing tumors [1]. The standard methods of radiation therapy are limited because any attempt to increase the total irradiation dose is associated with a high risk of severe post-radiation damage [2]. This necessitates new approaches to realize the main objective of radiation therapy, which is to increase the radiosensitivity of cancer cells. An increase in the effect of ionizing radiation can be achieved using radiosensitizers even without increasing the total irradiation dose [3,4].

Brown algae are an attractive source of biologically active compounds for researchers. They serve as raw materials for various therapeutic and prophylactic preparations, since they contain a number of bioactive substances, such as polysaccharides, mannitol, vitamins, macro- and microelements, polyphenols, iodine-containing organic compounds, and polyunsaturated fatty acids 

Phlorotannins are a type of phenolic metabolite found in brown algae. This group of phenolic compounds contains a large number of hydroxyl groups and chelates divalent metal ions; they have polymer structures and are highly soluble in water and firmly bound to proteins, polysaccharides, and other biopolymers. The monomer unit of phlorotannins is phloroglucinol (1,3,5-trihydroxybenzene). Based on the type of monomer bond, phlorotannins can be divided into four classes: fuhalols and phlorethols (ether bond), fucols (phenyl bond), fucophlorethols (ether and phenyl bonds), and eckols and carmalols (dibenzodioxin bond). Within each class, the binding of monomers to each other can occur at different positions of the phloroglucinol ring, which leads to the formation of structural isomers in addition to conformational ones. One species of algae is often known to produce phlorotannins with different structures and different degrees of polymerization [5].

We have previously shown that the aqueous ethanol extract of *Costaria costata* containing abundant phlorotannins possesses antitumor potential [6]. Moreover, phlorotannins from the same species of brown algae were found to be effective inhibitors of fucoidanase (enzyme hydrolyzed fucoidan) [7]. Since phlorotannins from brown algae are considered to have different types of biological activities and up to a certain concentration do not exert any toxic effect [8,9,10], they are of research interest as perspective bioactive agents for cancer therapy.

In the present work, we checked the hypothesis whether phlorotannins from *C. costata* inhibited cell viability and colony formation of human colorectal cancer cells as well as sensitized HT-29 and HCT 116 cells to X-ray radiation.

## 2. Results and Discussion

### 2.1. Characterization of the Phlorethols Isolated from Costaria costata

Phlorotannins constitute an extremely heterogeneous group of molecules, which differ by the types of bonds between the phloroglucinol units. Fucols have only phenyl bonds in the polymer molecule, phlorethols and fuhalols have ether bonds, and fucophlorethols have both of these bond types. The presence of a dibenzodioxin element in the structure is specific for eckols. Within each class, the binding of monomers to each other can take place at different positions of the phloroglucinol ring, resulting in the formation of structural isomers. The separation of longer, condensed phlorotannins is currently a challenge. [11].

The phlorotannins were isolated from an aqueous ethanol extract of the brown alga *C. costata.* Earlier the structural characteristics of phlorethols from *C. costata* were determined [7]. In the present work, NMR analysis showed that the phlorotannins of *C. costata* belong to the class of phlorethols (**CcPh**). The average molecular weight of **CcPh** was 2520 Da, as measured by ESI-MS in negative ionization mode. The degree of polymerization (DP) of the obtained phlorethols ranged from 12 to 25 phloroglucinol units with the most abundant phlorethols containing between 16 and 20 phloroglucinol units. [7] (Figures S1 and S2, and Table S1).

### 2.2. Bioactivity of the Phlorethols from C. costata

**Figure 1:**
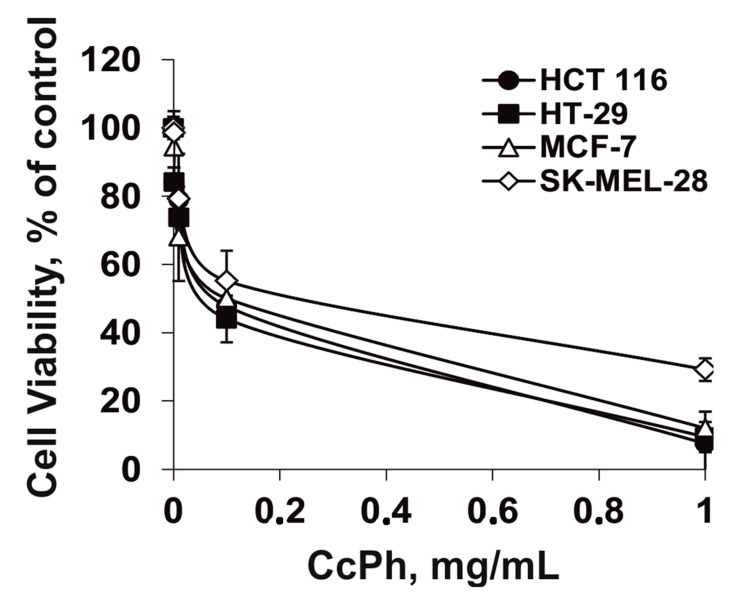
The cytotoxic activity of phlorethols from *C. costata* (**CcPh**) against colorectal carcinoma HT-29 and HCT 116 cells, breast cancer MCF-7 cells, and melanoma SK-MEL-28 cells. The cells were treated with **CcPh** at concentration of 0.001–1 mg/mL for 24 h. Cell viability was estimated using the MTS assay. Data are represented as the mean ± standard deviation (SD), as determined from triplicate experiments.

As a first step, the effect of phlorethols (**CcPh)** from brown alga *C. costata* (0–1000 µg/mL) on the viability of human colorectal carcinoma HT-29 and HCT 116 cell lines, breast cancer MCF-7 cells, and melanoma SK-MEL-28 cells was tested by MTS assay (Figure 1). The treatment of cells by **CcPh** was found to induce a concentration-dependent inhibition of cell’s viability. The inhibiting concentration of **CcPh** that caused a 50% reduction in cell viability (IC_50_) of HT-29 was 92 ± 2.8 µg/mL; HCT, 116–94 ± 3.08 µg/mL; MCF-7, 96 ± 3.3 µg/mL; and melanoma cells SK-MEL-28, 102 ± 4.8 µg/mL.

Recently, the cytotoxic activity of polyphenols’ fractions from different species of brown algae was investigated against pancreatic cancer cells Mia-PaCa-2, Panc-1, Panc-3.27, and BxPC-3 [12], human colorectal adenocarcinoma HT-29, Caco-2, breast carcinoma T-47D, MDA-MB-468 cell lines [13], human leukemia HL60 and THP-1, and prostate cancer PC3 cell lines [14]. The IC_50_ values for investigated polyphenols fractions were in a range from 80 to 200 µg/mL. It was suggested that the number of phenolic ring substituents and phenyl ether linkages as well as the reduction in pH values caused by an oxidation of hydroxyl groups in phenol polymer derivatives mainly influence their cytotoxic activity [15]. Since colorectal carcinoma cells HCT 116 and HT-29 gain resistance under radiation exposure [16,17], we chose them as model to check the hypothesis whether of phlorethols from brown alga *C. costata* is able to effectively sensitize these cells to radiation.

**Figure 2:**
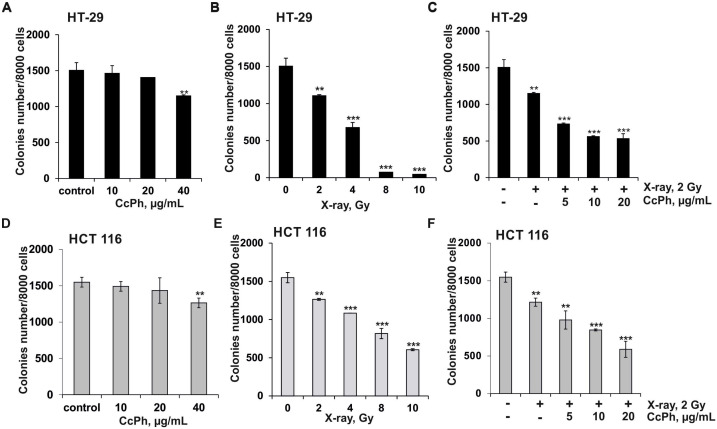
Anticancer and radiosensitizing effects of phlorethols from *C. costata* (**CcPh**) on colony formation in human colorectal carcinoma cells. HT-29 and HCT 116 cells (2.4 × 10^4^) were treated with (**A,D**) **CcPh** (10, 20, and 40 µg/mL) or (**B,E**) X-ray (2, 4, 8, and 10 Gy) or (**C,F**) a combination of X-ray radiation (2 Gy) and **CcPh** (5, 10, and 20 µg/mL) and subcultured onto Basal Medium Eagle BME soft agar and incubated for 2 weeks. The number of colonies was calculated using the ImageJ software bundled with 64-bit Java 1.8.0_112 (NIH, Bethesda, Maryland, USA). All experiments were repeated at least three times in each group. Results are expressed as the mean ± standard deviation (SD). The asterisk (*) indicates a significant decrease in the number of colonies of cancer cells treated with **CcPh** or X-ray compared to PBS-treated cells or **CcPh** in combination with X-ray compared to irradiated cells (** *p* < 0.01, *** *p* < 0.001).

Next, the effect of **CcPh** or X-ray radiation on colony formation in human colorectal cancer cells was determined using the soft agar assay. It was found that the **CcPh** alone decreased the number of colonies in HT-29 by 3%, 7%, and 24% at 10, 20, and 40 µg/mL, respectively and in HCT 116, by 4%, 7% and 18% at the same doses. (Figure 2A,D). It should be noted that **CcPh** did not influence the viability of normal mouse epidermal cells JB6 Cl41 at an effective concentration of 40 µg/mL even after 72-h of treatment (Figure S4).

The exposure of HT-29 by X-ray irradiation at doses of 2, 4, 8, and 10 Gy caused the inhibition of the colonies number by 26%, 55%, 95%, and 97%, respectively and HCT 116 by 18%, 30%, 47%, and 61% at the same doses (Figure 2B,E).

To investigate the radiosensitizing effect of phlorethols **CcPh**, HT-29 and HCT116 cells were treated with **CcPh** at non-toxic concentrations of 5, 10, and 20 µg/mL and exposed to a low dose of X-ray of 2 Gy, which alone slightly inhibited the viability or colony formation in cancer cells.

Under X-ray exposure, the number of colonies of HT-29 and HCT 116 cells were decreased by 24% and 21%, respectively, compared to the non-irradiated cells (control) (Figure 2C,F). The combinatory treatment with X-ray (2 Gy) and **CcPh** (5, 10, and 20 µg/mL) caused significant inhibition of colony formation of HT-29 by 28%, 39%, and 41%, respectively and HCT 116 by 15%, 24%, and 40%, respectively, compared to irradiated cells (Figure 2C,F).

To determine the type of combination effect of X-ray radiation with phlorethols **CcPh**, the combination index (CI) was calculated (Table 1). This revealed that the interactions of radiation and **CcPh** were mostly synergistic (CI < 0.7) [18]. **CcPh** strongly sensitized the HCT 116 cells to radiation as compared with HT-29 cells.

To the best of our knowledge, this is the first study on the radiosensitizing activity of phlorotannins from brown algae. Since polyphenols from brown algae possessed potent antioxidant activity, their radioprotective effect is a topic of interest of numerous investigations [19]. However there is evidence that several natural compounds are able to exert a dual mode of action after irradiation—radioprotective as well as radiosensitizing—depending on its dose and scheme of treatment [20]. For example, polyphenols of plant origin such as curcumin or resveratrol have been found to protect various systems against ionizing radiation and to sensitize cancer cells to radiation treatment [21].

## 3. Materials and Methods

### 3.1. Reagents

Phosphate buffered saline (PBS), L-glutamine, penicillin-streptomycin solution (10,000 U/mL, 10 µg/mL) were purchased from Sigma-Aldrich (St. Louis, MI, USA). The MTS reagent (3-(4,5-dimethylthiazol-2-yl)-5-(3-carboxymethoxyphenyl)-2-(4-sulfophenyl)-2H-tetrazolium) was purchased from Promega (Madison, WI, USA). The Basal Medium Eagle (BME), McCoy’s 5A Modified Medium (McCoy’s 5A), Minimum Essential Medium Eagle (MEM), trypsin, fetal bovine serum (FBS), and agar were purchased from ThermoFisher Scientific (Waltham, MA, USA). Organic solvents, inorganic acids, and salts were commercially obtained (Laverna, Moscow, Russia)

### 3.2. Cell Culture

Human colorectal carcinoma HT-29 (ATCC^®^ no. HTB-38™) and HCT 116 (ATCC^®^ CCL-247™) were cultured in McCoy’s 5A medium, supplemented with 10% FBS and 1% penicillin-streptomycin solution; breast cancer MCF-7 (ATCC^®^ no. HTB-22™) or melanoma SK-MEL-28 (ATCC^®^ no. HTB-72™) cells were maintained in MEM medium with 10% FBS and 1% penicillin-streptomycin solution. The cell cultures were maintained at 37 °C in humidified atmosphere containing 5% CO_2_. Every 3–4 days, the cells were detached with 0.25% trypsin/0.05 M EDTA for 1–3 min and 10–20% of the harvested cells were transferred to a new flask containing fresh complete culture media.

### 3.3. Isolation of the Phlorethol Fraction (**CcPh**) from C. costata

The brown alga *C. costata* (Turn) Saund (order Laminariales) was collected in Peter the Great Bay, Sea of Japan in July 2016. The isolation, separation, and structural characterization of phlorethols were conducted as previously reported [7].

### 3.4. Preparation of the Phlorethol Fraction (**CcPh**) for Bioassays

**CcPh** was dissolved in sterile PBS to prepare stock concentrations of 10 mg/mL. Cells were treated with serially diluted phlorethols (culture medium used as diluent) to give the intended final concentrations (5–1000 µg/mL). The vehicle control was the cells treated with an equivalent volume of PBS for all presented experiments.

### 3.5. MTS Assay

HT-29 and HCT 116 or MCF-7 and SK-MEL-28 cells (1.0 × 10^4^) were seeded in 200 µL of complete McCoy’s 5A/10% FBS or MEM/10% FBS media, respectively, and incubated for 24 h at 37 °C in 5% CO_2_ incubator. The attached cells were treated by phlorethols **CcPh** at concentrations ranging from 0 to 1000 µg/mL for an additional 24 h. Subsequently, the cells were incubated with 15 µL MTS reagent for 3 h, and the absorbance of each well was measured at 490/630 nm using PowerWave XS Microplate Reader (BioTek, Winooski, VT, USA). All samples were tested in triplicate.

### 3.6. Soft Agar Assay

To estimate the effect of phlorethols **CcPh** on colony formation (phenotype expression), human colorectal carcinoma cells HT-29 and HCT 116 (2.4 × 10^4^) were treated with PBS (control) or phlorethols **CcPh** (10, 20, and 40 µg/mL) in 1 mL of 0.3% BME agar containing 10% FBS, 2 mM l-glutamine, and 25 µg/mL gentamicin. The cultures were maintained in a 37 °C, 5% CO_2_ incubator for 14 days, and the cell colonies were scored using a Motic microscope AE 20 (XiangAn, Xiamen, China) and ImageJ software bundled with 64-bit Java 1.8.0_112 (NIH, Bethesda, MD, USA).

### 3.7. Cell Irradiation Assay

Human colorectal carcinoma cells HT-29 and HCT 116 were exposed to X-ray radiation using an XPERT 80 X-ray system (KUB Technologies, Inc., Milford, CT, USA). The absorbed dose of radiation was measured by a DRK-1 X-ray radiation clinical dosimeter (Axelbant LLK, Moscow, Russia).

To determine the sensitivity of HT-29 or HCT 116 cells to radiation, the cells (5.0 × 10^5^) were exposed to X-ray at a dose rate from 2 to 10 Gy and recovered at 37 °C in a 5% CO_2_ incubator for 3 h. The cells were harvested with 0.25% trypsin/0.05 M EDTA solution and subjected to the “Soft agar assay” as described above.

To determine the radiosensitizing activity of phlorethols, HT-29 or HCT 116 cells (5.0 × 10^5^) were exposed to 2 Gy of X-ray and incubated for 3 h. Then, cells were treated with either PBS (control) or phlorethols **CcPh** (5, 10, and 20 μg/mL) for an additional 24 h. The cells were harvested and used for the “Soft agar assay” as described above.

### 3.8. Combination Index (CI) Calculation

The calculations of drug concentration/X-ray irradiation dose-effect were performed by CompuSyn software version 1.0 (ComboSyn, Inc., Paramus, NJ, USA) using the median effect method described by Chou and Talalay [22].

### 3.9. Data Analysis

All assays were performed in at least three independent experiments. Results are expressed as the mean ± standard deviation (SD). The Student’s t-test was used to evaluate the data with the following significance levels: * *p* < 0.05, ** *p* < 0.01, *** *p* < 0.001.

## 4. Conclusions

This study evaluated the in vitro anticancer and radiosensitizing effects of phlorethols (**CcPh**) from *C. costata* on human colorectal carcinoma HCT 116 and HT-29 cell lines. **CcPh** at non-toxic concentrations was found to significantly inhibit the colony formation in HT-29 and HCT 116 alone and in combination with X-ray irradiation. The combinatory effect of radiation and **CcPh** was synergistic (combination index CI < 0.7). The molecular mechanism of this action requires further research. In future studies, we plan to determine the effects of polyphenols on the detoxification of enzymes, the activation of endogenous protective systems, and the repair of DNA strand breaks induced by X-ray exposure.

## Figures and Tables

**Table 1:** Combination index (CI) for phlorethols—X-ray irradiation interactions.

Concentration of CcPh, µg/mL	Dose of X-ray, Gy	Combination Effect, % of Control		Combination Index, CI *	
		HT-29	HCT 116	HT-29	HCT 116
2.5	2	40	30	0.67879 ± 0.024	0.58038 ± 0.012
5	2	52	37	0.61559 ± 0.03	0.48424 ± 0.035
10	2	63	45	0.58341 ± 0.027	0.43194 ± 0.042
20	2	65	62	0.66600 ± 0.021	0.33167 ± 0.037

* The Combination Index (CI) is the quantitative measure of the degree of interaction between different treatments. CI values in the range of 0.9–1.1 indicate additive effect; and CI values greater than 1.1, antagonism. CI values in the range from 0.9 to 0.7 indicate slight synergism; and CI values less than 0.7, synergism. CI values were calculated according to the Chou and Talalay mathematical model for drug interactions using the CompuSyn software version 1.0 on the basis of the results of soft agar assay. Data represent mean CI calculated from three independent experiments ± standard deviations.

## Supplementary Materials

The following are available online, Figure S1: ^13^C-NMR spectra of the **CcPh** fraction, Figure S2: HMBC NMR spectra of the **CcPh** fraction, Figure S3: HSQC NMR spectra of the **CcPh** fraction, Table S1: The data of elemental compositions, monoisotopic masses and ions of the phlorethol **CcPh** fraction, Figure S4: The effect of phlorethols from *C. costata* (**CcPh**) on viability of normal mouse epidermal cells JB6 Cl41.
