# Supplementary material for: In Vitro Anticancer and Radiosensitizing Activities of Phlorethols from the Brown Alga Costaria costata
Source: Molecules. 2020 Jul 14;25(14):3208. doi: 10.3390/molecules25143208 (PMC7396992; doi:10.3390/molecules25143208)
Supplement: Supplementary file 1 [file molecules-25-03208-s001.pdf]

# Supplementary material

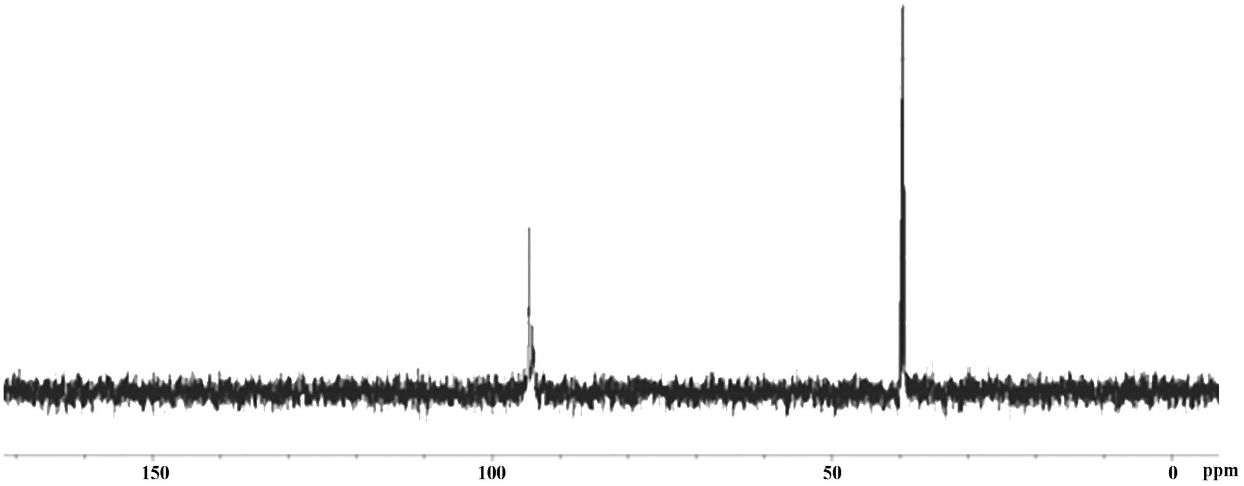


**Figure S1.** $^{13}$C-NMR spectra of the **CcPh** fraction.

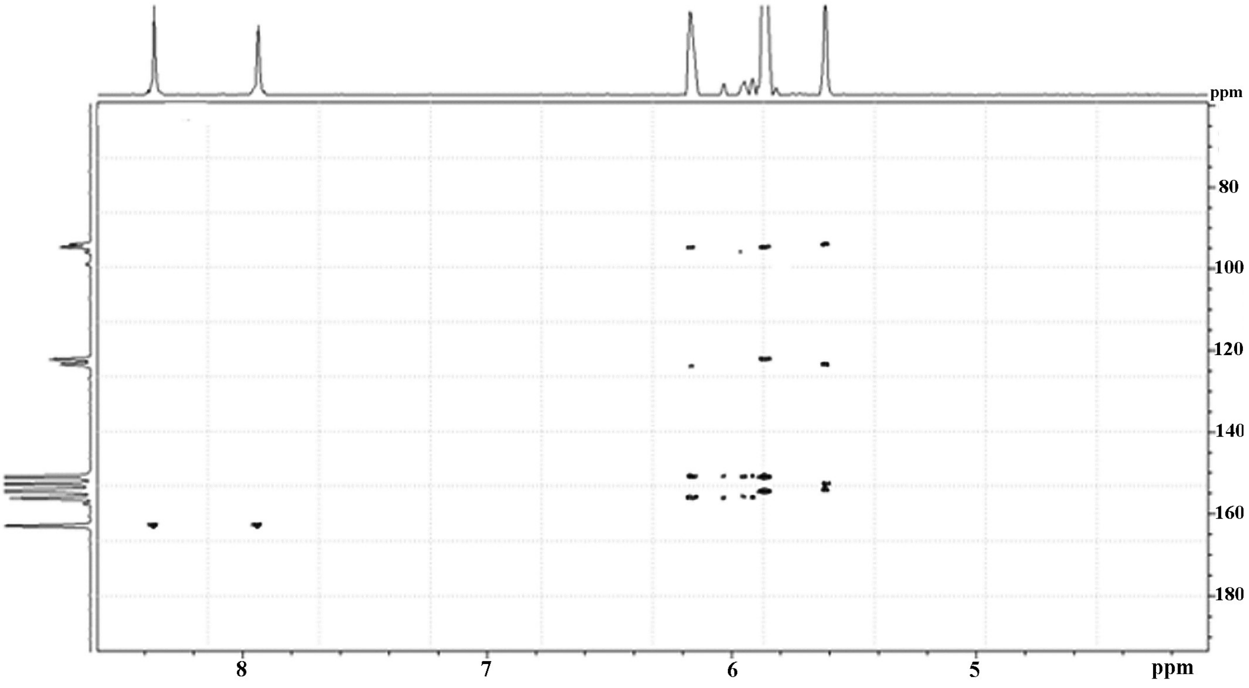


**Figure S2.** HMBC NMR spectra of the **CcPh** fraction.

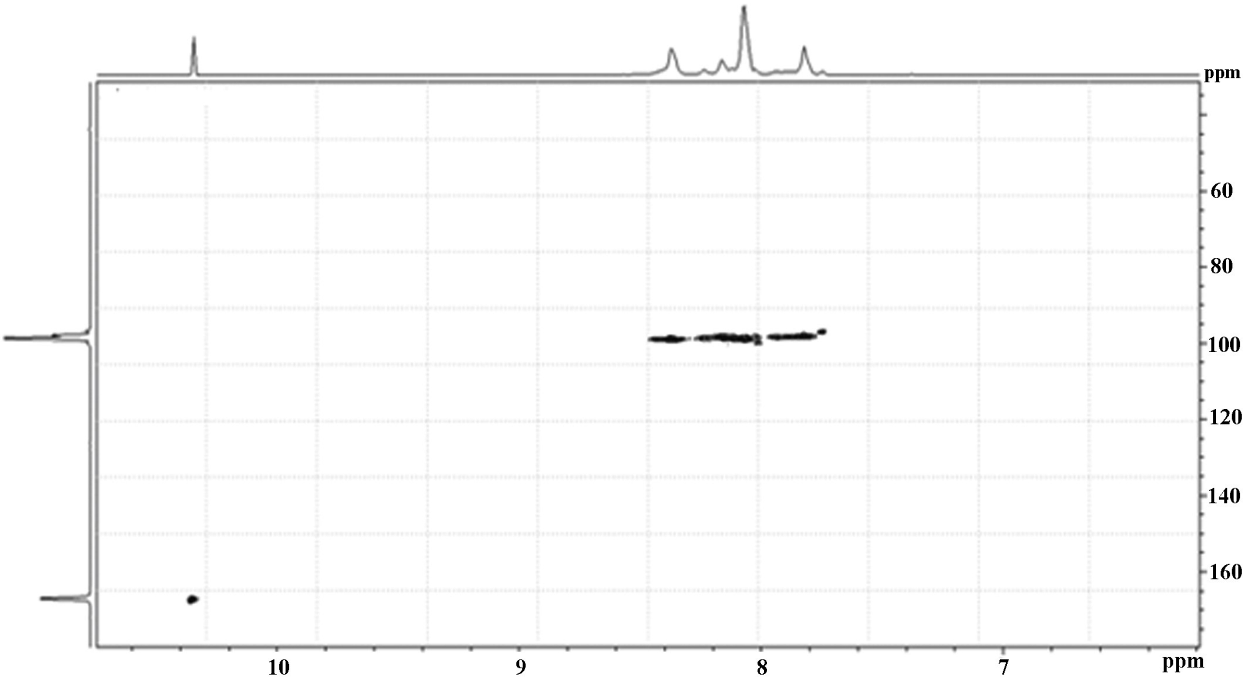


**Figure S3.** HSQC NMR spectra of the **CcPh** fraction.

**Supplementary Table S1.** The data of elemental compositions, monoisotopic masses and ions of the phlorethol **CcPh** fraction detected in negative ion mode HRMS at each degree of polymerization (DP), representing the predominant charge state detected under the experimental conditions described. The strength of MS signals expressed as a percentage (%) of intensity (100%=600).

| DP | Elemental Composition | Monoisotopic Massa (Da) | Masses of Observed Phlorethol Ions (*m*/*z*) | | Signal Strength |
|---|---|---|---|---|---|
| | | | $[M-H]^-$ | $[M-2H]^{-2}$ | |
| 12 | $C_{72}H_{50}O_{36}$ | 1490.2081 | 1489.2072 | - | 20 |
| 13 | $C_{78}H_{54}O_{39}$ | 1614.2242 | 1613.2237 | - | 15 |
| 14 | $C_{84}H_{58}O_{42}$ | 1738.2402 | - | 868.1180 | 20 |
| 15 | $C_{90}H_{62}O_{45}$ | 1862.2563 | - | 930.1263 | 15 |
| 16 | $C_{96}H_{66}O_{48}$ | 1986.2723 | - | 992.1359 | 50 |
| 17 | $C_{102}H_{70}O_{51}$ | 2110.2883 | - | 1054.1331 | 20 |
| 18 | $C_{108}H_{74}O_{54}$ | 2234.3044 | - | 1116.1437 | 70 |
| 19 | $C_{114}H_{78}O_{57}$ | 2358.3204 | - | 1178.1608 | 20 |
| 20 | $C_{120}H_{82}O_{60}$ | 2482.3365 | - | 1240.1602 | 50 |
| 21 | $C_{126}H_{86}O_{63}$ | 2606.3525 | - | 1302.1773 | 15 |
| 22 | $C_{132}H_{90}O_{66}$ | 2730.3686 | - | 1364.1867 | 40 |
| 23 | $C_{138}H_{78}O_{69}$ | 2854.3846 | - | 1425.1981 | 10 |
| 25 | $C_{150}H_{102}O_{75}$ | 3102.4167 | - | 1550.2111 | 10 |

- Absence of singly charged ions in the mass-spectrum.

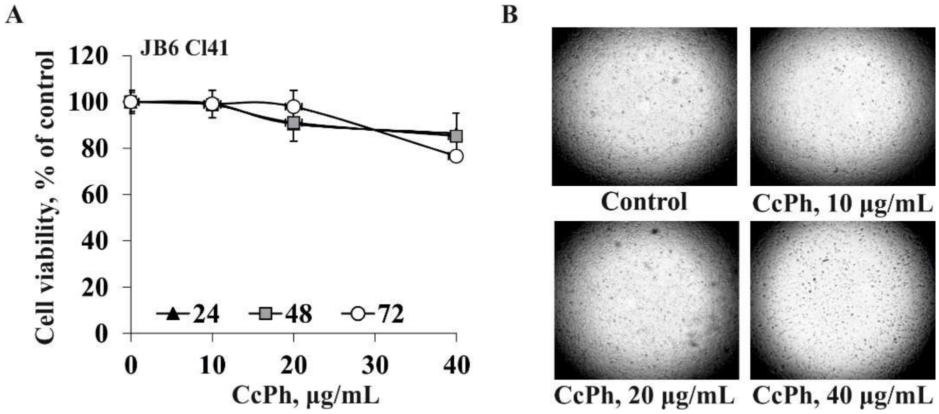


**Figure S4.** The effect of phlorethols from *C. costata* (**CcPh**) on viability of normal mouse epidermal cells JB6 Cl41. (**A**) Cells ($8\times10^3$ cells/ 200µl) were treated with **CcPh** (10–40 µg/mL), or PBS, as a negative control, for 24, 48, and 72 h. The cytotoxicity was determined using MTS assay. Data are represented as the means ± SD as determined from triplicate experiments. (**B**) The representative image of viable JB6 Cl41 cells after 72 h of incubation with **CcPh** made under a microscope with the aid of the ImageJ software program. The magnification of photos is ×10.
